# Characteristics of Skin Lesions Determine the Therapeutic Response of Facial Café Au Lait Macules Laser Therapy

**DOI:** 10.1111/jocd.70062

**Published:** 2025-02-11

**Authors:** Yuanzhi Liu, Wanshan Yang, Yuan Ding, Bin Yang, Zhenfeng Liu

**Affiliations:** ^1^ Laser and Dermatology Surgery Center Dermatology Hospital of Southern Medical University Guangzhou Guangdong China

**Keywords:** café au lait macules, laser therapy, morphologic characteristics, picosecond laser, Q‐switched alexandrite laser

## Abstract

**Background:**

The efficacy of laser treatment for facial café au lait macules (CALMs) is random.

**Aim:**

To compare the response of different characteristics of CALMs skin lesions to laser treatment.

**Patients/Methods:**

In this single‐center retrospective case series, patients with café au lait macules who received laser treatment between 2015 and 2022 at our clinic were reviewed. A total of 319 consecutive patients were eligible and were treated with either a 755‐nm‐alexandrite picosecond laser or a quality‐switched 755‐nm‐alexandrite laser. Observers were blinded to the final patient groups. Efficacy was graded according to four levels of treatment response: poor (Grade 1, 0%–25% improvement), fair (Grade 2, 26%–50% improvement), good (Grade 3, 51%–75% improvement), and excellent (Grade 4, 76%–100% improvement). Treatment effects evaluated as Grades 2–4 were considered effective.

**Results:**

Of the 319 patients, excellent and good responses were observed in 80 (25.08%) and 66 (20.69%) cases, respectively. Fifty‐two patients (16.30%) displayed an outcome of Grade 2 (26%–50% improvement), whereas 121 (37.93%) cases showed an outcome of Grade 1 (0%–25% improvement). The overall treatment effective rate (Grades 2–4) was 62.07%. Binary logistic regression analysis showed a significant association of therapeutic efficacy with lesion distribution (segmental vs. non‐segmental CALMs) and lesion border (irregular vs. regular) (*p* < 0.05 for both).

**Conclusions:**

Segmental and irregular border CALMs tended to respond well to laser therapy. Clinicians can leverage these characteristics to predict efficacy and manage patient expectations more effectively.

## 1 Introduction

Café au lait macules (CALMs) are benign epidermal pigmentation disorders, with a prevalence of approximately 15% in the general population and 2% of all newborns [1, 2]. The lesions can appear as round, oval, or irregular light‐to‐dark‐brown macules or patches ranging from 1 to 200 mm. Multiple or isolated lesions may be present; multiple lesions can be associated with neurofibromatosis or other genodermatoses. Histopathology shows increased epidermal melanin levels without melanocytic proliferation [3]. The dermoscopic features of CALMs included visible brown pigment patches characterized by clear borders and varying sizes. Under reflectance confocal microscopy (RCM), the features of CALMs were marked by increased pigment content in the basal layer of the skin lesion, numerous highly refractive particles, and no significant alterations in the superficial dermis [4].

Most CALMs are benign, and when identified, the greatest concern is often cosmetic [5]. Especially when the lesions occur on exposed parts such as the face, they can negatively affect patients' lives, including social interaction and mental health. In clinical practice, there appears to be a wide variability in morphology [6], some have smooth, regular borders, and others have more jagged irregular edges [4, 5]. CALMs have also been observed to be either isolated or segmental distributed clinically [7]. This may be due to the fact that melanocytes arise from the neural crest, and the cephalic neural crest gives rise to most of the craniofacial skeleton and other facial tissues such as nerve ganglia in addition to melanocytes [7]. However, there has been little exploration, to our knowledge, as to how morphologic characteristics might affect treatment response [1].

There is no uniformly successful modalities for the treatment of CALMs [8]. Traditional modalities such as cryotherapy, electrofulguration, and surgical excision are associated with scar formation and dyspigmentation [9]. To overcome these drawbacks, laser treatments on the basis selective photothermolytic theory were developed [2], including 532 nm quality‐switched (Q‐switched) Nd:YAG laser, Q‐switched ruby laser, Q‐switched alexandrite laser, erbium‐doped yttrium aluminum garnet laser, and picosecond laser [9]. As melanin particles show high absorption of laser energy at wavelengths of 532 and 755 nm, lasers at these wavelengths have been used more frequently [10]. Owing to the high incidence of purpura with 532 nm wavelengths, 755‐nm‐alexandrite laser has been considered to have high clearance with minimal adverse effects [11]. The picosecond laser, which was initially developed for tattoo removal, has raised much interest owing to its excellent promise in treating hyperpigmented disorders, compared with the Q‐switched alexandrite laser [12]. This may be because the picosecond laser has a shorter pulse duration than the Q‐switched laser, which can generate a greater photomechanical effect with a tensile strength that exceeds the tissue's ultimate tensile stress [12]. To date, few studies have compared the efficacy of picosecond and nanosecond lasers in the treatment of CALMs.

The effect of laser treatment is random. Studies have shown that the morphological characteristics of CALMs can predict efficacy to some extent. CALMs with irregular borders are easier to remove than those without [1]. However, little is known about whether the distribution of CALMs can affect the response to laser treatment. Therefore, we performed a retrospective analysis to compare the efficacy between the 755‐nm‐alexandrite picosecond and Q‐switched 755‐nm‐alexandrite lasers for treating CALMs, as well as differences in therapeutic responses of lesions with different distributions.

## 2 Materials and Methods

This study was approved by the Medical Ethics Committee of the Dermatology Hospital of Southern Medical University. Written informed consent was obtained from all patients or their legal guardians.

Medical records of 319 patients with CALMs who received treatment between 2015 and 2022 at the local center were reviewed. Patients were treated with either a 755‐nm‐alexandrite picosecond laser (PicoSure; Cynosure Inc., Westford, MA) or a Q‐switched 755‐nm‐alexandrite laser (Accolade; Cynosure Inc., Westford, MA). A total of 156 patients and 163 patients were treated with Q‐switched 755 nm laser and 755 nm picosecond laser, respectively. The efficacy, safety, and factors associated with efficacy were analyzed.

Demographic and clinical data reviewed included sex, age, age of onset, distribution of lesions, border of lesions, Fitzpatrick skin type, number of treatments, efficacy, adverse reactions, and relapse. All patients received 1 to 6 treatments, with intervals ranging from 1 to 16 months. Decisions to retreat were based primarily on clinical responses and the patients' wishes. Digital photographs were taken before each treatment, and treatment parameters were recorded after each treatment.

Two independent dermatologists who were blinded to morphologic and distribution category respectively assessed efficacy. Therapeutic efficacy was graded as follows: poor, Grade 1, 0%–25% improvement; fair, Grade 2, 26%–50% improvement; good, Grade 3, 51%–75% improvement; and excellent, Grade 4, 76%–100% improvement. Treatments evaluated as Grades 2–4 were considered effective. Treatment effect was evaluated primarily by whether the area of the lesion was reduced and/or whether the color of the lesion was lightened. An excellent improvement was considered if the lesion was barely visible to the naked eye, and the remaining grading criteria were based on the subjective judgment of the dermatologist on the extent of lesion area and color improvement. After the round of independent assessments, any discrepancies between the two dermatologists were resolved through discussion to reach a consensus.

Analyses were performed using SPSS (version 25.0; IBM Corp., Armonk, NY, USA). Binary logistic regression analysis was used to evaluate the influence of factors on treatment outcomes. The dependent variable was treatment efficacy. Independent variables included the following items: sex, age of onset, age at time of laser treatment, Fitzpatrick skin type, lesion characteristics, laser type, and treatment times. The odds ratio (OR) was calculated to estimate relative risk and the significance level of the maximum likelihood ratio test (*p* < 0.05). Chi‐squared test was used to evaluate the effects of treatment times and different laser types on the incidence of adverse reactions.

## 3 Results

Among the 319 patients considered, excellent and good responses were observed in 80 (25.08%) and 66 (20.69%) cases, respectively. Fifty‐two patients (16.30%) displayed an outcome of Grade 2 (26%–50% improvement), whereas 121 (37.93%) cases showed an outcome of Grade 1 (0%–25% improvement).

**FIGURE 1:**
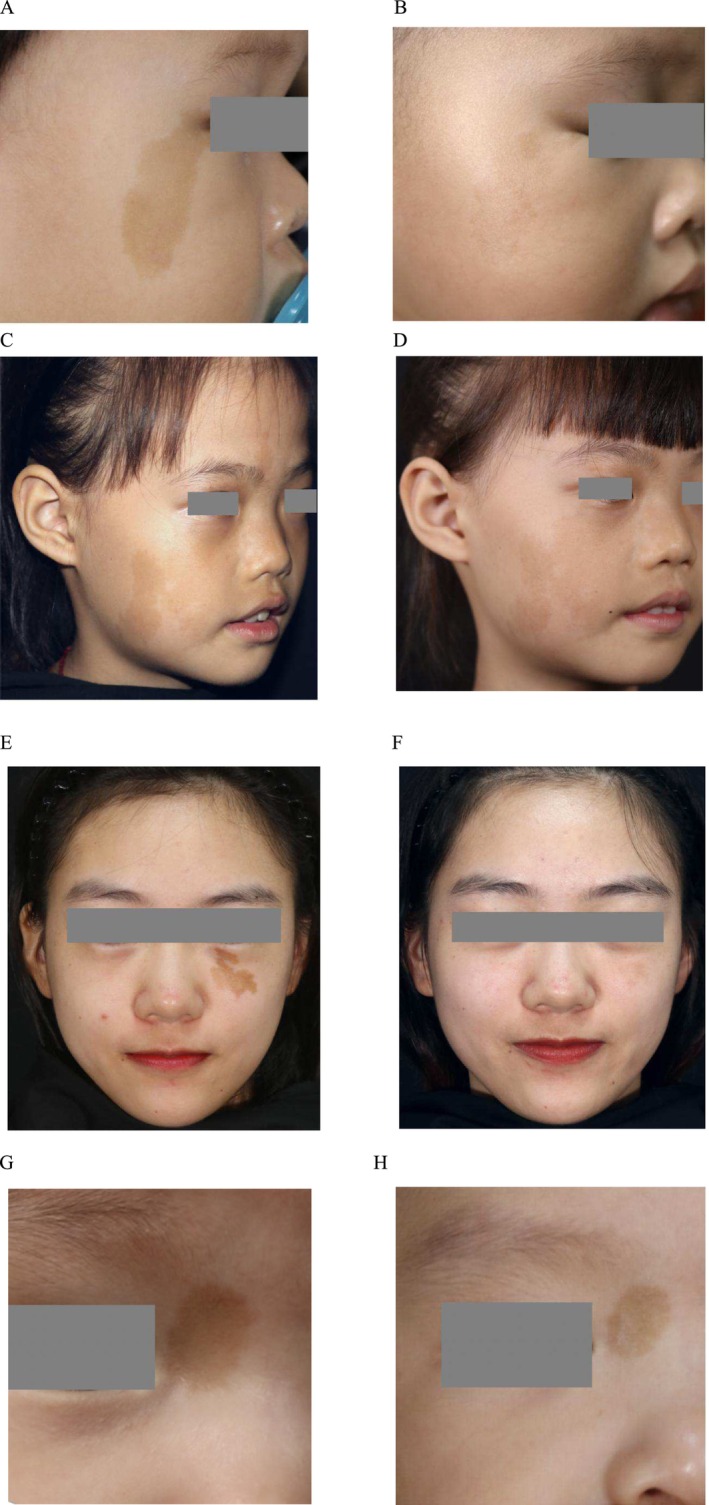
Treatment effect of facial CALMs with different distribution and borders. (A) Facial segmental CALMs before treatment. (B) Three‐month follow‐up after 1 ps, 755 nm laser treatment showed excellent clearance. (C) Facial segmental CALMs before treatment. (D) Eleven‐month follow‐up after one Q‐switched 755 nm laser treatment showed fair clearance. (E) Facial CALMs with irregular borders before treatment. (F) four‐month follow‐up after 1 ps, 755 nm laser treatment showed excellent clearance. (G) Facial non‐segmental CALMs with regular borders before treatment. (H) Six‐month follow‐up after 2 ps, 755 nm laser treatments showed poor clearance.

An improvement of Grades 2–4 was observed in 74.2% of patients with segmental CALMs, whereas this improvement was observed only in 54.15% of patients with non‐segmental CALMs. Binary logistic regression analysis revealed significant differences in treatment efficacy between segmental and non‐segmental CALMs (*p* < 0.05), as well as between irregular and regular lesion borders (*p* < 0.001). Responses of segmental CALMs and irregularly edged lesions were significantly more favorable than those of non‐segmental CALMs and regularly edged lesions, respectively (Figure 1). The average ages of onset and at the time of treatment were 2.24 and 14.82 years (range, 4 months–60 years), respectively. However, the treatment efficacy neither varied significantly with the age of onset, age at treatment, lesion distribution, and treatment method, nor differed significantly between the 755‐nm‐alexandrite picosecond laser and Q‐switched 755‐nm‐alexandrite laser (69.23% vs. 55.21%). (Table 1).

**TABLE 1:** Patients' demographic and clinical characteristics.

Characteristic	Number of patients	Coefficient	OR	*p*
Gender				
Male	95	−0.470	0.625	0.124
Female	224			
Mean age of onset	319	−0.026	0.975	0.312
Mean age at time of laser treatment	319	0.005	1.005	0.709
Fitzpatrick skin type				
III	117	−0.545	0.580	0.193
IV	202			
Border of CALMs				
Irregular	213	−1.767	0.171	< 0.001
Smooth	106			
Color of CALMs				
Uneven	128	0.022	1.022	0.949
Even	191			
Distribution of CALMs				
Non‐segmental	219	0.795	2.214	0.011
Segmental	100			
No. of treatments per patient				
≤ 2	278	0.417	1.517	0.311
> 2	41			
Laser type				
Q‐switched	156	−0.718	0.488	0.071
Picosecond	163			
Treatment effectiveness				
Effective	198	62.07%		NA
Ineffective	121	37.93%		

Abbreviations: CALMs, café au lait macules; NA, not applicable; OR, odds ratio.

This study showed that adverse reactions after laser treatment include hypopigmentation, postinflammatory hyperpigmentation, mottled discoloration, and an expanded lesion area. Chi‐squared tests showed that rates of adverse reactions were positively correlated with the number of treatments but did not differ significantly between the different lasers (Tables 2 and 3). Of the 127 patients followed‐up for more than 6 months after treatment, 21 (16.53%) experienced recurrences.

**TABLE 2:** Laser type and adverse reactions.

Laser type	No.	Adverse reactions (%)					df	*χ*2	*p*
		Hyperpigmentation	Hypopigmentation	Mottled discoloration	Expanded lesion area	Total			
Q755	156	$10\;\left(6.41\%\right)$	$9\;\left(5.77\%\right)$	$49\;\left(31.41\%\right)$	$4\;\left(2.56\%\right)$	72 (46.15%)	1	0.007	0.933
Ps755	163	$33\;\left(20.25\%\right)$	$3\;\left(1.84\%\right)$	$30\;\left(18.40\%\right)$	$10\;\left(6.13\%\right)$	76 (46.62%)			

Abbreviation: No, number of patients.

**TABLE 3:** Treatment times and adverse reactions.

Treatment times	Adverse reactions (%)	df	*χ*2	*p*
1	75 (38.27%)	5	23.095	<0.001
2	42 (51.85%)			
3	17 (68.00%)			
4	11 (84.62%)			
5	2 (100.00%)			
6	2 (100.00%)			

## 4 Discussion

Although harmless, the cosmetic concern of CALMs is troublesome to patients, and most patients seek medical help. Prior to 1983, when the selective photothermolysis theory was introduced, traditional treatments of CALMs included chemical peeling, cryotherapy, mechanical grinding, electrofulguration, and surgical operations [13]. Since then, laser treatment has been considered the most effective therapy for CALMs. Laser therapy involves the rapid vaporization of melanin upon absorption of laser energy. However, responses to various laser treatments among lesions with different morphological characteristics are inconsistent. In theory, 532 nm (frequency‐doubled Nd:YAG) wavelengths are most appropriate for epidermal lesions, followed by 755 nm (alexandrite) wavelengths. Owing to the high incidence of purpura with 351–532 nm wavelengths, a 755 nm (alexandrite) laser was used to treat patients in this study [14]. Studies have shown favorable safety profiles and no permanent adverse reactions of both picosecond treatments and Q‐switched nanosecond laser treatments [10, 15, 16, 17].

Hyperpigmented macules or patches that might be confused with CALMs include pigmentary mosaicism (linear nevoid hyperpigmentation), Becker melanosis (without obvious hypertrichosis), and postinflammatory hyperpigmentation. Smaller lesions may resemble lentigines or acquired melanocytic nevi, whereas larger lesions may be confused with relatively flat congenital melanocytic nevi [18]. The differential diagnosis between CALMs and other hyperpigmented dermatoses can be established on the basis the characteristics of skin lesions, dermoscopic examination, histopathological biopsy, and the assessment of multisystemic involvement.

The neural crest gives rise to melanocytes and several other cell types. As a transient structure present during embryonic development, the neural crest is composed of highly multipotent progenitor cells, characterized by populations of determined precursors, as well as heterogeneous and multipotent cells, capable of giving rise to different phenotypes [7]. This may explain the wide phenotypic variation in syndromes associated with CALMs.

In our study, facial segmental CALMs tended to respond better to laser treatment than non‐segmental CALMs, whose mechanism remains unclear and requires further studies. The pathogenesis of segmental CALMs also remains unclear, which may relate to cutaneous nerve distribution and pigment mosaic. This subtype of CALMs should be differentiated from pigmentary demarcation lines that present as bilateral, well‐defined, homogenous, hyperpigmented patches.

In the present study, binary logistic regression analysis after the removal of confounding factors revealed that CALMs with irregular borders tended to respond well to laser treatment, which is consistent with the findings of previous reports [1, 19]. Our results also showed that treatment outcomes of 755‐nm‐alexandrite nanosecond and 755‐nm‐alexandrite picosecond lasers did not significantly differ, consistent with findings of a randomized, split‐lesion clinical trial conducted by Cen et al. [12]. The adverse reactions to laser treatment include hypopigmentation, postinflammatory hyperpigmentation, mottled discoloration and an expanded lesion area [3, 20]. These adverse events can be attributable to laser‐induced inflammation, which increases pigmentation. During a 6‐month follow‐up, the recurrence rate was 16.53%, which fell in the low range of 0%–67%, as reported previously [21].

A limitation of this study was the lack of an objective evaluation of treatment efficacy. Besides, the main limitation of this study is the retrospective nature and lack of randomization. Thus, a multicenter, randomized, parallel controlled trial is required to confirm our findings.

In conclusion, facial segmental CALMs and lesions with irregular borders tended to respond well to laser treatment. The 755‐nm‐alexandrite nanosecond and 755‐nm‐alexandrite picosecond lasers were equally effective for CALMs. The incidence rate of adverse reactions was positively correlated with the number of treatments. Therefore, treatment decisions should be made on the basis of the clinical response and patients' wishes. The findings of this study may help physicians predict outcomes and manage patient expectations accordingly.

## Author Contributions

Drs. Yuanzhi Liu and Zhenfeng Liu had full access to all of the data in the study and take responsibility for the integrity of the data and the accuracy of the data analysis. Concept and design: Bin Yang and Zhenfeng Liu. Acquisition, analysis, or interpretation of data: All authors. Drafting of the manuscript: Yuanzhi Liu and Wanshan Yang. Critical revision of the manuscript for important intellectual content: Zhenfeng Liu and Bin Yang. Statistical analysis: Wanshan Yang, Yuan Ding, and Yuanzhi Liu. Supervision: Bin Yang and Zhenfeng Liu.

## Ethics Statement

The authors confirm that the ethical policies of the journal, as noted on the journal's author guidelines page, have been adhered to and the appropriate ethical review committee approval has been received.

## Conflicts of Interest

The authors declare no conflicts of interest.

## Data Availability

The data that support the findings of this study are available from the corresponding author upon reasonable request.
